# Spontaneous calcified cerebral emboli: a comprehensive review and proposed diagnostic criteria

**DOI:** 10.3389/fneur.2024.1401820

**Published:** 2024-07-17

**Authors:** Spiro Menounos, Walid Matar

**Affiliations:** ^1^Department of Neurology, St George Hospital Kogarah, Sydney, NSW, Australia; ^2^St George Clinical School, University of New South Wales, Sydney, NSW, Australia

**Keywords:** calcified cerebral emboli, stroke, embolic stroke, cerebral infarction, calcified aortic valve

## Abstract

Spontaneous calcified cerebral emboli (SCCE) secondary to aortic valve calcification are a rare and underreported cause of acute ischaemic stroke. Only five cases of SCCE secondary to bicuspid aortic valve calcification have been reported in the literature. This review includes a unique case example of acute ischaemic stroke secondary to SCCE, as the first manifestation of a calcified bicuspid aortic valve. This is the first clinical case of calcified cerebral emboli (CCE) associated with borderzone infarction (‘cortical ribbon sign’). Whilst previously assumed that most CCE are secondary to iatrogenic causes, recent literature suggests the majority of CCE are spontaneous and clinically silent. Despite CT imaging widely considered the ‘gold standard’ for diagnosis, CCE are frequently misdiagnosed and missed entirely. Misdiagnosis of CCE may have catastrophic consequences due to the high risk of recurrence and missed opportunity to prevent neurological disability and death. This review presents a revised CCE diagnostic criteria, using evidence that has emerged over the last decade to create both Compulsory (Major) and Supporting (Minor) criteria. Current CCE management is not evidence based and remains largely speculative. SCCE may be the first manifestation of cardiac or vascular disease and diagnosis should trigger aggressive treatment of emboligenic sources. Future epidemiological studies, analysing symptomatic and asymptomatic SCCE patients, would be beneficial in providing accurate quantification of disease burden. Other future research directions include exploring intracranial stenting for CCE revascularisation and cerebral intravascular lithotripsy.

## 1 Introduction

Spontaneous calcified cerebral emboli (SCCE) are underreported and often remain clinically silent (1). The incidence of calcified cerebral emboli (CCE) has been demonstrated between 2.7%–5.9% of all acute ischaemic stroke (AIS) presentations, suggesting CCE is far more common than previously assumed (2, 3). Whilst initial studies speculated that CCE were mainly iatrogenic (4), more recent literature has revealed that the majority of CCE are associated with spontaneous cerebral infarction (5, 6). Aortic valve calcification (AVC) is the most common emboligenic source (2). Despite CCE distinct appearance, 27% are misdiagnosed and 9% are missed entirely. Misdiagnosis of CCE may have catastrophic consequences due to the high risk of recurrence (43%) (2). Unsurprisingly, patients with SCCE experience worse clinical outcomes and higher mortality rates (7, 8). This review presents a revised CCE diagnostic criteria using evidence that has emerged over the last decade. Management of SCCE is not evidence based, with data limited to mainly case series level evidence.

This review presents a unique case of AIS secondary to SCCE, as a first manifestation of a calcified bicuspid aortic valve (BAV) in a 54 year-old man. Computed Tomography (CT) imaging demonstrated a 6 mm CCE in the left M1 segment of middle cerebral artery (MCA). On magnetic resonance imaging (MRI), this corresponded with left hemispheric ‘cortical ribbon sign’, due to borderzone infarction and is the first case of CCE associated with borderzone infarction. The patient underwent aortic valve replacement (AVR) and was medically managed with aspirin. Over the next 3 months, the patient made good recovery, with minor persisting neurological deficits.

## 2 Case example

A 54 year-old man was found collapsed on the floor at home, last seen well 24 h previously. Prior to this event he was fully independent. His medical history included epilepsy, dyslipidaemia, bipolar affective disorder, anxiety, depression and previous intravenous drug use. His usual medications were carbamazepine 400 mg BD, quetiapine XR 2,000 mg daily, atomoxetine 100 mg daily, escitalopram 10 mg daily, levetiracetam 500 mg BD, rosuvastatin 10 mg daily and buprenorphine/naloxone 32/8 mg daily. There was no past history of atrial fibrillation, rheumatic heart disease or endocarditis. There was no family history of cardiovascular disease. In the emergency department, his National Institutes of Health Stroke Scale (NIHSS) was 10 for dysphasia, right facial upper motor neuron deficit and right side hemiparesis; with fluctuating neurological deficits. Examination revealed an ejection systolic murmur. There were no clinical stigmata of infective endocarditis.

**Figure 1:**
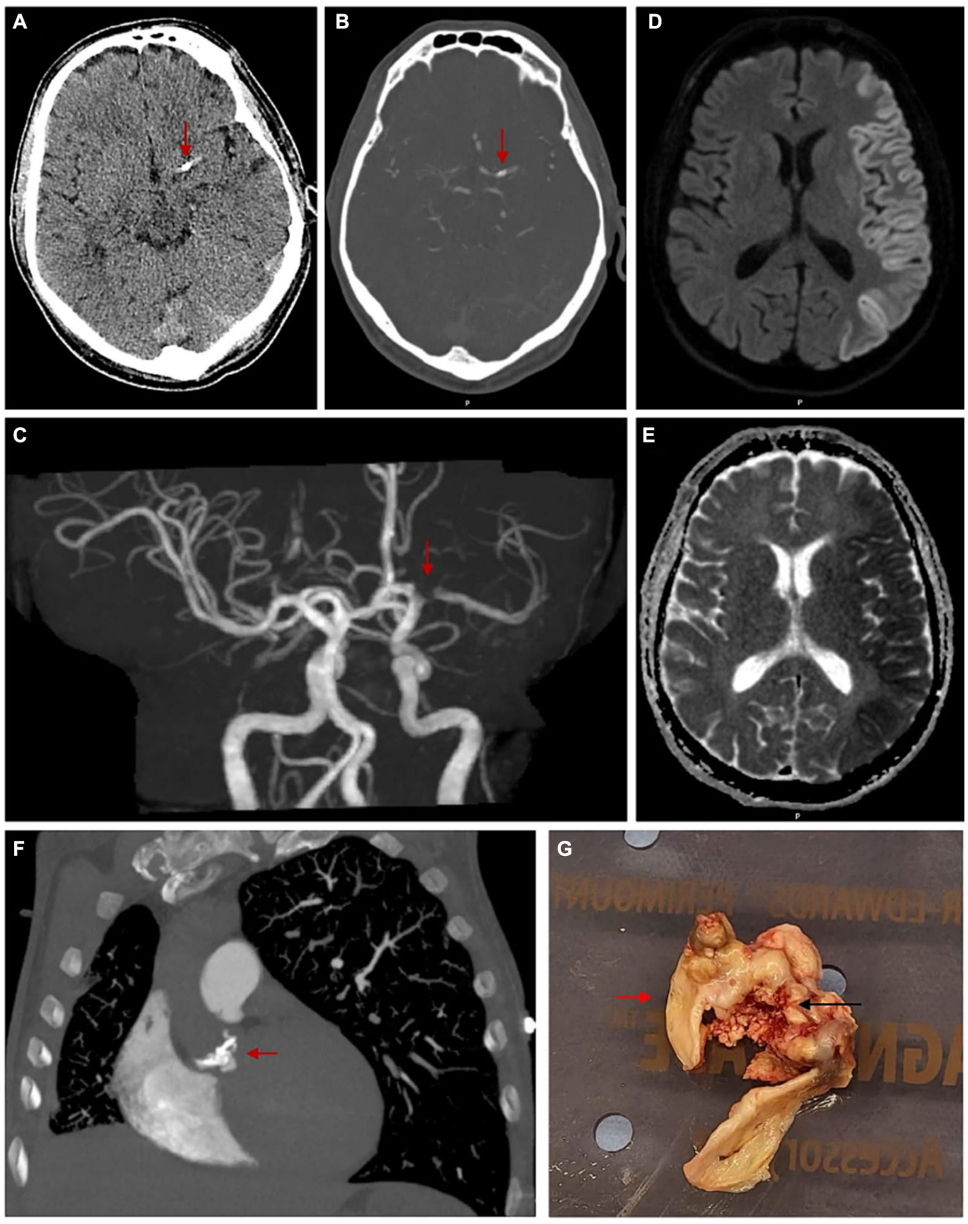
Images demonstrating a calcified cerebral embolism in the M1 segment of left MCA, left hemispheric cerebral cortex diffusion restriction and nodular calcification of bicuspid aortic valve leaflets. **(A)** Axial view of unenhanced CT brain with red arrow marking a calcified focus measuring 6 mm in the vicinity of proximal left MCA. **(B)** Axial view of CT brain angiogram with red arrow marking a calcified focus involving the proximal M1 segment of left MCA. **(C)** 3D reconstruction of CT brain angiogram with red arrow marking a filling defect involving the M1 segment of left MCA, corresponding with site of calcific cerebral embolism. **(D)** MRI brain diffusion-weighted images in axial view demonstrating left hemispheric cortical hyperintensity or ‘Cortical Ribbon Sign’. **(E)** Corresponding MRI brain apparent diffusion coefficient images in axial view demonstrating left hemispheric cortical hypointensity. **(F)** CT pulmonary angiogram in coronal view with red arrow marking multiple densities/calcifications over the bicuspid aortic valve. **(G)** Macroscopic photo taken intra-operatively with red arrow marking normal bicuspid aortic valve and black arrow marking dystrophic calcification over bicuspid aortic valve.

Extensive haematological investigations including blood count, electrolytes, lipid profile, antiphospholipid syndrome antibody screen and serology studies yielded no significant results. CT brain non-contrast and perfusion demonstrated a 6 mm oval shaped CCE in the M1 segment of left MCA (Figures 1A–C). Diffusion-weighted MRI demonstrated borderzone infarction, with left hemispheric cortical hyperintensity (‘cortical ribbon sign’) and corresponding hypointensity on the apparent diffusion coefficient images (Figures 1D,E). This is the first case of CCE which has revealed ‘cortical ribbon sign’. Transoesophageal echocardiogram revealed bicuspid aortic valves (type I, RCC/LCC subtype) with a large vegetation, moderate aortic regurgitation, moderate-to-severe aortic stenosis and no patent foramen ovale. CT chest demonstrated aortic valve densities/calcifications, in keeping with vegetations (Figure 1F). CT angiogram demonstrated no evidence of flow-limiting stenosis, occlusion or calcification in the anterior and posterior circulation in the neck. Microbial investigations for infective endocarditis were all consistently negative.

In this current case, the CCE source was calcified BAV. The source of calcification was thought to be secondary to a combination of a factors including the bicuspid aortic valve, dyslipidaemia and previous history of intravenous drug use. Subsequently, the patient was treated for culture-negative endocarditis and underwent AVR, where a large ulcerated calcific lesion was noted on the leaflets (Figure 1G). Histopathological evaluation of this lesion revealed dystrophic calcification, fibrosis, patchy oedema and chronic inflammation. Treatment involved an initial one-off aspirin dose of 300 mg, then regular 100 mg daily. Over the next 5 months, the patient made good recovery with persisting mild neurological deficits.

## 3 Discussion

### 3.1 Prevalence and aetiology of spontaneous calcified cerebral emboli

SCCE are an underreported cause of AIS and often asymptomatic (1). Studies have reported incidences between 2.7–5.9% of all AIS, suggesting SCCE is more common than previously assumed (2, 3). Early reports speculated that CCE were mainly iatrogenic/non-spontaneous, secondary to manual dislodgement from valve surgery, left heart catheterisation or atheromatous aortic and carotid artery manipulation (4). More recent studies demonstrate that the majority of CCE are associated with spontaneous cerebral infarction (2, 5, 6). Statistical analysis of 70 CCE cases, revealed 86% occurred spontaneously, whilst only 14% of cases were iatrogenic (2).

Emboligenic sources of SCCE include calcified aortic and mitral valves (9–11), as well as atheromatous plaques in the arch of the aorta (12), carotid and vertebral arteries (12–14) and brachiocephalic trunk (15). Of these, AVC has been reported as the most common (2). Frequently, the origin of SCCE are difficult to establish. One retrospective multicentre study of 40 CCE patients, reported an unknown embolisation source in 37.5% of cases (7). Similarly, Mosqueira and colleagues could not identify SCCE sources in 33.3% of patients (5). Only 30 cases of SCCE secondary to AVC have been published in the literature (Supplementary Table 1).

### 3.2 Aortic valve calcification and spontaneous calcified cerebral emboli

Aortic valve sclerosis (thickening and calcification) is common, being detected in 26% of individuals ≥ 65 years old (16). This prevalence increases with age, as aortic valve sclerosis is present in 48% of those ≥85 years old (17). BAV is the most common congenital heart disease and accelerates development of AVC (18). This is due to abnormal bicuspid valve geometry, that predisposes to degenerative calcification and increases the risk of calcific dislodgement (19, 20). On retrospective analysis, BAV patients with cerebral or retinal infarctions had lower CHA_2_DS_2_-VASc scores and less traditional risk factors, when compared to patients with tricuspid aortic valves (19). Thunstedt and colleagues suggest that in SCCE patients with an absence of cerebral macroangiopathy or cardiovascular disease risk, one should consider BAV as an embolic source, especially in young patients (9). Amongst the 30 cases of SCCE secondary to AVC available in the literature, 5 associated with BAV were identified (Supplementary Table 1).

Autopsy case series have reported an association between AVC and spontaneous calcific emboli. Holley and colleagues examined 165 autopsy patients with AVC and found 22% (37 cases) had evidence of spontaneous calcific embolisation (1). Of the 45 emboli identified in 37 patients, common sites included the coronary, retinal and renal arteries. Of note, only 1 patient had a CCE and only 4/45 emboli presented with clinical symptoms of infarction (1 renal, 3 coronary). A further autopsy series also reported 28 cases of systemic calcific emboli in 81 patients with AVC, but only one-fifth occurred spontaneously (21). This imbalance between the rare occurrence of clinically symptomatic CCE and frequent CCE identification in asymptomatic individuals on autopsy studies, has several implications (22, 23). Firstly, it suggests the majority of CCE could be too small for radiological diagnosis and may be overlooked or misdiagnosed. Secondly, ischaemic events caused by CCE may be very subtle and mistakenly attributed to associated causes such as poor cerebral perfusion or cerebral small vessel disease. Thirdly, only the symptomatic minority of CCE patients receive treatment, as the overwhelming asymptomatic majority remain unnoticed. Despite autopsy studies demonstrating an associated between AVC and spontaneous calcific emboli, this relationship has not been identified in non-CCE patients. One study prospectively investigating 402 consecutive patients with non-CCE cardioembolic stroke, only identified one patient with calcified aortic stenosis (24).

### 3.3 Diagnostic methods of calcified cerebral emboli

Unenhanced CT and CT angiography are imaging modalities of choice in visualising CCE and appear as small oval shaped hyperdense lesions, that lie within cerebral arterial tract vasculature. Case reports thus far have revealed two main CCE distribution patterns. The first distribution pattern is far more common, involving 1–2 focal CCE located centrally within major intracranial arteries such as the MCA. The second pattern, initially referred to as the ‘salted pretzel sign’, includes many small CCE located in the pial arteries, resulting from a ‘calcified shower’ (13). Amongst the 30 published cases of SCCE secondary to AVC, only 3 cases were associated with >2 CCE (Supplementary Table 1). MRI is less useful and proves difficult when identifying CCE, but is essential in determining extent of ischaemia. Echocardiogram plays a crucial role investigating the origins of CCE, through revealing aortic valve morphology (tricuspid vs. BAV) and presence of AVC (25).

Despite CT widely regarded as the ‘gold standard’ for CCE identification, they are frequently misdiagnosed or ignored. One study revealed 27% of CCE on initial unenhanced CT brain interpretation were misdiagnosed and as much as 9% were missed entirely (2). In this study, included cases of CCE were assumed to be a gross underestimation of real disease prevalence, as cases were identified on the basis of search terms alone, rather than by visually reviewing thousands of CT scans. When using CT angiogram, several studies note the ‘false patency sign’ as one of the common pitfalls for CCE identification (26–28). This results from the isodense appearance of CCE with respect to iodine contrast, simulating vessel patency. To avoid this pseudopatency phenomenon, Nouh et al. (28) suggest consideration of low dose CT angiography when clinical suspicion of CCE remains high, despite false patency sign. More broadly, to overcome the diagnostic challenge of CCE, clinicians/radiologists require greater education and awareness of common radiological features associated with CCE.

When comparing CCE to ‘regular’ non-calcified emboli, CCE have more round/ovoid shapes and are more attenuated with an average Hounsfield units (HU) of 160, compared to the 50–70 HU of regular emboli (2). A 35 patient analysis reported an average CCE density of 428 HU, with the lowest being 89 HU (6). Whilst no consensus definition of CCE density thresholds has been established, several studies have used thresholds of >70 HU (2, 6), >90 HU (8) and >130 HU (7) as inclusion criteria for CCE diagnosis. Features of intracranial atheromatous stenosis that can be differentiated from CCE, include the eccentric shape, position not in the vessel lumen and location rarely distal to the internal carotid artery terminus (6). Cerebral vessel calcifications are more commonly tubular/linear in shape, contrasting the round/ovoid appearance of CCE (8). Other CCE mimics to rule out include hyperdense artery sign, small haemorrhage, sequelae from past infections, normal calcified structures such as the choroid plexus, calcified granulomas or cavernomas and neurocysticercosis (10). The MCA has consistently been demonstrated to be the most common location of CCE and localisation may be another assisting factor in supporting CCE diagnosis (2, 5–7). Out of 30 cases of SCCE secondary to AVC identified in this review, the MCA was involved in the overwhelming majority (Supplementary Table 1).

### 3.4 Diagnostic criteria of calcified cerebral emboli

CCE misdiagnosis may have catastrophic consequences, due to the high risk of recurrence and missed opportunity to prevent future embolisation, neurological disability and death. The recurrence rate of embolic infarction in CCE patients is 43% and significantly more common than conventional thromboembolism (2). Unsurprisingly, patients with AIS secondary to CCE experience worse clinical outcomes and higher mortality rates (7, 8). There are no guidelines to assist clinicians/radiologists in diagnosing SCCE from different emboligenic sources. The only SCCE diagnostic criteria within the literature, has been suggested by Khetarpal et al. (25), and solely focuses on calcified aortic valve stenosis as the origin of embolisation.

Using evidence that has emerged over the last decade, this review presents a revised CCE diagnostic criteria incorporating both Compulsory (Major) and Supportive (Minor) criteria (Table 1). To facilitate greater diagnosis of asymptomatic SCCE, which are thought to be responsible for the overwhelming majority, as part of the Compulsory criteria, CCE are required to be located in clinically relevant areas for symptomatic patients only. Furthermore, the Supportive criteria takes into account various emboligenic sources of CCE and predispositions to AVC such as BAV. Other components of the Supportive criteria are clinical/radiological features which favour the diagnosis of CCE, such as previous CCE, localisation to the MCA and presence of chronic kidney disease (CKD). Comorbidities such as CKD are associated with development of valvular/vascular calcification (29), and several reports have demonstrated the presence of CCE in CKD patients (10, 30–33).

**Table 1:** Diagnostic criteria for spontaneous calcified cerebral emboli.

**Compulsory (Major) criteria**
A small circular/oval lesion that is hyperdense (>70 HU) on unenhanced CT and localises to the corresponding cerebral arterial tract vasculature within the cerebral parenchyma AND For symptomatic CCE only, the hyperdense lesion is located in a clinically relevant area AND Absence of recent procedures such as cardiac catheterisation, calcified aortic/mitral valve replacement or carotid manipulation
**Supportive (Minor) criteria**
Embolic source present such as carotid/aorta atherosclerosis, aortic/mitral valve calcification Presence of BAV if no embolic source is found Previous CCE CCE localised to MCA Chronic kidney disease

HU, Hounsfield unit; CT, Computed tomography; CCE, Calcified cerebral emboli; BAV, bicuspid aortic valve; MCA, middle cerebral artery.

### 3.5 Management of calcified cerebral emboli

Management of CCE can be separated into hyperacute stroke treatment and secondary prevention. Hyperacute treatment involves tissue plasminogen activator (tPA) and/or endovascular thrombectomy (ET). Outcomes from tPA are conflicting, with positive (2, 12) and negative (34, 35) results in the literature. The general consensus is that tPA is less effective in CCE patients, as the fibrin-degrading properties have little effect on calcium and cholesterol content (5, 8). Studies suggest a potential benefit from tPA, involving dissolution of fibrin-rich thrombus surrounding CCE, potentially making it more favourable to ET (7, 8). The first case of successful ET recanalisation in AIS secondary to CCE, was reported in 2016 (36). Despite technical success, the clinical outcome was disappointing with an unchanged NIHSS of 15 and post-procedure imaging demonstrating established infarction. From an endovascular treatment perspective, CCE pose a technical challenge, due to unique biomechanical properties increasing retrieval difficulty and vascular injury (37). Compared to non-CCE patients, CCE patients undergoing ET had significantly longer procedure times (58 vs. 75 min, respectively) and increased complications, including intracranial haemorrhage, thought to be secondary to greater technical difficulty and applied traction for CCE removal (8).

One of the first case series investigating ET safety and efficacy in CCE patients, demonstrated 0% reperfusion rate using a thrombo-aspiration technique (38). A few years later, Bruggeman et al. (8) contrastingly reported successful reperfusion in 44% of CCE patients with functional independence in 29%. In this study, ET included stent retrieval, thrombo-aspiration or a combination of both techniques. Reperfusion rates and functional outcomes were comparable between CCE patients who received tPA prior to ET and those who did not, supporting the notion that tPA has relatively low efficacy for CCE (8). Grand et al. (6) recently validated these results, performing the first systematic review and meta-analysis investigating ET effectiveness in CCE large vessel occlusion. This retrospective multicentric national analysis identified 35 patients, where 57% obtained successful reperfusion and 28% achieved good clinical outcome (Modified Rankin scale ≤2). These findings were included in individual patient-based meta-analysis to reach 136 CCE cases treated with ET, where reperfusion rates and good clinical outcomes were 50 and 29%, respectively (6).

Rigorous methodological assessment of all emboligenic sources such as the aortic/mitral valves, arch of the aorta, brachiocephalic trunk and carotid and vertebral arteries, will guide secondary CCE prevention. Early reports of CCE secondary to AVC, initially recommended medical management with antiplatelet therapy (25, 39). One case study, identified CCE secondary to a mobile string-like thrombus attached to AVC (39). Interestingly, the thrombus gradually regressed and disappeared after commencement of antiplatelet therapy and upon discontinuation, the string-like thrombus was again noted. The benefit of AVR in the context of CCE has not yet been investigated and currently there is no conclusive evidence to support replacement. Given the high frequency of CCE recurrence (2), the current consensus amongst many authors supports AVR as ‘prophylactic source control’ in removing emboligenic origins to prevent further CCE (9, 20, 22, 40–42). No documented cases exist in the literature of CCE recurrence following AVR. Oliveira-Filho et al. (42) describe calcific emboli recurrence in a patient with CCE secondary to a calcified BAV, where warfarin therapy was initially pursued instead of AVR. The patient subsequently underwent AVR and remained stroke free on 2-year follow up. Xu et al. (43) highlights that calcified BAV as an embolic source, probably exceeds medical management and surgery should be considered in AVC patients with cerebrovascular events and absence of other embolic sources. Ultimately, the decision for patients to undergo AVR for ‘prophylactic source control’ in the context of CCE secondary to AVC is complex. Consideration should be made to the high CCE recurrence rate vs. surgical risks of AVR and prosthetic valve morbidity.

### 3.6 Future directions

Additional clinical data including large case series and controlled trials are needed when determining the best hyperacute revascularisation approach and secondary prevention strategies for CCE patients. Small sample sizes remain a significant limitation in the evidence for CCE management, preventing sufficient power for analysis to create recommendations for first-line treatment options. In the absence of evidence based therapy and clear treatment guidelines, clinicians are left to speculate on the most appropriate management pathway for CCE patients. Further epidemiological studies examining the incidence of SCCE in both symptomatic and asymptomatic patients with AVC, would be of great benefit. Given the literature suggests that clinically silent CCE are in-fact the majority (1), these epidemiological studies would provide more accurate quantification of true CCE disease burden. Greater awareness and diagnosis of spontaneous asymptomatic CCE will facilitate earlier implementation of secondary prevention, prior to CCE recurrence. This will also allow analysis of asymptomatic patients with CCE secondary to AVC, a unique sub-population with little-to-no current data in the literature, to determine efficacy of secondary prevention strategies such as prophylactic AVR.

Other future research directions include the possibility of intracranial stenting for CCE revascularisation and cerebral intravascular lithotripsy (IVL). Potts et al. (44) reported 2 cases where intracranial stenting was successfully used in CCE patients with AIS after ET failed. Despite restoring good flow following stent deployment, the role of intracranial stenting for CCE revascularisation remains unclear. Additional evidence is required before comparisons can be made with ET, given the risks of vessel injury and rupture. Additionally, IVL being a novel technique adapted from nephrolithiasis therapy, is regarded as a breakthrough in calcified carotid artery stenosis treatment and could possibly be explored in CCE management. IVL uses pulsatile sonic pressure waves to disrupt and fracture arterial wall calcification, without harming normal surrounding tissue (45). Given the irregular, stiff and sharp-edged nature of CCE that impair adhesion to traditional ET techniques, IVL using a peripheral lithotripsy catheter may potentially alter CCE shape to be more favourable for ET. When considering the frequently documented technical difficulties of ET in CCE patients (2, 8), exploring IVL as an adjunct therapy may improve ET reperfusion rates but more research is needed to better define its role.

Other future research directions include exploring the relationship between the location of vascular cerebral topography and neurological symptom severity in patients with SCCE. For AIS secondary to non-CCE, this relationship has already been established, where patients with anterior cerebral artery infarction have a favourable short term prognosis and show a unique clinical profile. Defining such a relationship in SCCE patients would be of value however requires additional clinical data (46). A significant limitation of this current study includes the restricted number of SCCE clinical cases published in the literature. In the future, this may be ameliorated with greater awareness of SCCE and increased publication of clinical data.

## 4 Conclusion

SCCE is an underreported cause of AIS and more common than previously assumed. Recent literature suggests the majority of CCE are spontaneous and clinically silent. CCE are frequently misdiagnosed or missed entirely. This review presents a revised CCE diagnostic criteria, using evidence that has emerged over the last decade. Current CCE management is not evidence based and remains largely speculative. Future studies analysing SCCE incidence in symptomatic and asymptomatic patients would be beneficial in quantifying CCE disease burden.

## Author contributions

SM: Conceptualization, Writing – original draft, Writing – review & editing. WM: Conceptualization, Writing – original draft, Writing – review & editing.

## References

[1] Holley KE Bahn RC McGoon DC Mankin HT. Spontaneous calcific embolization associated with calcific aortic stenosis. Circulation. (1963) 27:197–202. doi: 10.1161/01.CIR.27.2.197, PMID: 14173487

[2] Walker BS Shah LM Osborn AG. Calcified cerebral emboli, a "do not miss" imaging diagnosis: 22 new cases and review of the literature. AJNR Am J Neuroradiol. (2014) 35:1515–9. doi: 10.3174/ajnr.A3892, PMID: 24651819 PMC7964438

[3] Bardon M Hanson J O'Brien B Naeem A. Calcified cerebral emboli: incidence and implications. J Med Imaging Radiat Oncol. (2018) 62:499–503. doi: 10.1111/1754-9485.12730, PMID: 29665308

[4] Vernhet H Torres GF Laharotte JC Tournut P Bierme T Froment JC. Spontaneous calcific cerebral emboli from calcified aortic valve stenosis. J Neuroradiol. (1993) 20:19–23. PMID: 8492172

[5] Mosqueira AJ Canneti B Martinez Calvo A Fernandez Armendariz P Seijo-Martinez M Pumar JM. Calcified cerebral embolism: a 9-case series and review of the literature. Neurologia. (2022) 37:421–7. doi: 10.1016/j.nrl.2019.04.004 34785159

[6] Grand T Dargazanli C Papagiannaki C Bruggeman A Maurer C Gascou G. Benefit of mechanical thrombectomy in acute ischemic stroke related to calcified cerebral embolus. J Neuroradiol. (2022) 49:317–23. doi: 10.1016/j.neurad.2022.02.006, PMID: 35183595

[7] Maurer CJ Dobrocky T Joachimski F Neuberger U Demerath T Brehm A. Endovascular Thrombectomy of calcified emboli in acute ischemic stroke: a multicenter study. AJNR Am J Neuroradiol. (2020) 41:464–8. doi: 10.3174/ajnr.A6412 32029470 PMC7077917

[8] Bruggeman AAE Kappelhof M Arrarte Terreros N Tolhuisen ML Konduri PR Boodt N. Endovascular treatment for calcified cerebral emboli in patients with acute ischemic stroke. J Neurosurg. (2021) 135:1402–12. doi: 10.3171/2020.9.JNS201798, PMID: 33799302

[9] Thunstedt DC Muller K Kupper C Becker R Mehr M Heck S. Juvenile stroke caused by calcific bicuspid aortic valve: a case report. Clin Neurol Neurosurg. (2020) 195:106079. doi: 10.1016/j.clineuro.2020.106079, PMID: 32663736

[10] Taoussi R Khattab H Jadib A Daki A Bendahou H Sabiri M. Transient ischemic attack due to multiple spontaneous calcified embolus of the cerebral arteries on a calcified mitral and aortic stenosis. Radiol Case Rep. (2022) 17:2899–901. doi: 10.1016/j.radcr.2022.05.043, PMID: 35733951 PMC9207546

[11] Mahajan A Goel G Banga V Chatterjee A. Beware of brain pearl-virtually missed a large vessel occlusion guided by CT perfusion. Neurol India. (2022) 70:816–7. doi: 10.4103/0028-3886.344606, PMID: 35532676

[12] Kavanagh EC Fenton DM Heran MK Lapointe JS Nugent RA Graeb DA. Calcified cerebral emboli. AJNR Am J Neuroradiol. (2006) 27:1996–9. PMID: 17032882 PMC7977882

[13] Christian BA Kirzeder DJ Boyd J Laing J Gash JR. Showered calcific emboli to the brain, the 'salted pretzel' sign, originating from the ipsilateral internal carotid artery causing acute cerebral infarction. Stroke. (2009) 40:e319–21. doi: 10.1161/STROKEAHA.108.538009 19286589

[14] Tardy J Da Silva N Glock Y Larrue V. Neurological pictures. Stroke with calcium emboli related to a calcified stenosis of internal carotid artery. J Neurol Neurosurg Psychiatry. (2008) 79:1273–4. doi: 10.1136/jnnp.2007.140749, PMID: 18940991

[15] Moustafa RR Antoun NM Coulden RA Warburton EA Baron JC. Stroke attributable to a calcific embolus from the brachiocephalic trunk. Stroke. (2006) 37:e6–8. doi: 10.1161/01.STR.0000195211.76192.ed, PMID: 16306455

[16] Stewart BF Siscovick D Lind BK Gardin JM Gottdiener JS Smith VE. Clinical factors associated with calcific aortic valve disease. Cardiovascular health study. J Am Coll Cardiol. (1997) 29:630–4. doi: 10.1016/S0735-1097(96)00563-3 9060903

[17] Otto CM Lind BK Kitzman DW Gersh BJ Siscovick DS. Association of aortic-valve sclerosis with cardiovascular mortality and morbidity in the elderly. N Engl J Med. (1999) 341:142–7. doi: 10.1056/NEJM199907153410302 10403851

[18] Thiene G Rizzo S Basso C. Bicuspid aortic valve: the most frequent and not so benign congenital heart disease. Cardiovasc Pathol. (2024) 70:107604. doi: 10.1016/j.carpath.2024.107604 38253300

[19] Huntley GD Michelena HI Thaden JJ Alkurashi AK Pislaru SV Pochettino A. Cerebral and retinal infarction in bicuspid aortic valve. J Am Heart Assoc. (2023) 12:e028789. doi: 10.1161/JAHA.122.028789, PMID: 36942747 PMC10122894

[20] O'Donoghue ME Dangond F Burger AJ Suojanen JN Zarich S Tarsy D. Spontaneous calcific embolization to the supraclinoid internal carotid artery from a regurgitant bicuspid aortic valve. Neurology. (1993) 43:2715–7. doi: 10.1212/WNL.43.12.2715, PMID: 8255488

[21] Soulie P Caramanian M Soulie J Bader JL Colcher E. Calcareous embolism of calcified orificial lesions of the left heart. Arch Mal Coeur Vaiss. (1969) 62:1657–84. PMID: 4987969

[22] Natale F Aronne L Credendino M Siciliano A Allocca F Weizs SH. Which is the correct management of patients with asymptomatic severe calcific aortic stenosis after symptomatic spontaneous calcium cerebral embolism? J Cardiovasc Med. (2011) 12:428–9. doi: 10.2459/JCM.0b013e328344bcc7, PMID: 21346590

[23] Kapila A Hart R. Calcific cerebral emboli and aortic stenosis: detection of computed tomography. Stroke. (1986) 17:619–21. doi: 10.1161/01.STR.17.4.619, PMID: 3738941

[24] Pujadas Capmany R Arboix A Casanas-Munoz R Anguera-Ferrando N. Specific cardiac disorders in 402 consecutive patients with ischaemic cardioembolic stroke. Int J Cardiol. (2004) 95:129–34. doi: 10.1016/j.ijcard.2003.02.007, PMID: 15193810

[25] Khetarpal V Mahajan N Madhavan R Batra S Mopala P Sagar A. Calcific aortic valve and spontaneous embolic stroke: a review of literature. J Neurol Sci. (2009) 287:32–5. doi: 10.1016/j.jns.2009.07.018, PMID: 19712938

[26] Uneda A Kanda T Suzuki K Hirashita K Yunoki M Yoshino K. Acute cerebral artery occlusion by a calcified Embolus with false patency sign on computed tomographic angiography. J Stroke Cerebrovasc Dis. (2017) 26:e5–7. doi: 10.1016/j.jstrokecerebrovasdis.2016.09.029, PMID: 27789151

[27] Yogendrakumar V Patro S Dowlatshahi D Stotts G Iancu D. Calcified embolus mimics patent middle cerebral artery on CT angiogram. Pract Neurol. (2017) 17:307–9. doi: 10.1136/practneurol-2017-001631 28659455

[28] Nouh A Wrubel G Balabhadra A Hou Y. Calcified basilar artery Embolus with false-patency sign on computed tomography angiogram. Stroke. (2023) 54:e423–4. doi: 10.1161/STROKEAHA.123.043790, PMID: 37522574

[29] Marwick TH Amann K Bangalore S Cavalcante JL Charytan DM Craig JC. Chronic kidney disease and valvular heart disease: conclusions from a kidney disease: improving global outcomes (KDIGO) controversies conference. Kidney Int. (2019) 96:836–49. doi: 10.1016/j.kint.2019.06.025, PMID: 31543156

[30] Bugnicourt JM Bonnaire B Lepage L Garcia PY Lefranc M Godefroy O. Stroke due to spontaneous calcified cerebral embolus as presenting feature of calcified aortic stenosis. J Mal Vasc. (2008) 33:106–9. doi: 10.1016/j.jmv.2008.02.004, PMID: 18455337

[31] Nakayama T Wakino S Itoh H. Calcified cerebral embolism due to aortic valve calcification. Clin Exp Nephrol. (2020) 24:1084–5. doi: 10.1007/s10157-020-01923-7, PMID: 32594373

[32] Kobayashi S Iizuka K Takekawa H Suzuki K. A case of calcified cerebral emboli during hemodialysis. Neurol Therapeut. (2022) 39:727–30.

[33] Katsamakis G Lukovits TG Gorelick PB. Calcific cerebral embolism in systemic calciphylaxis. Neurology. (1998) 51:295–7. doi: 10.1212/WNL.51.1.295 9674827

[34] Halloran JI Bekavac I. Unsuccessful tissue plasminogen activator treatment of acute stroke caused by a calcific embolus. J Neuroimaging. (2004) 14:385–7. doi: 10.1111/j.1552-6569.2004.tb00270.x, PMID: 15358964

[35] Okazaki S Sakaguchi M Sugiyama Y Ooe H Kitagawa K Sakoda S. Ineffective thrombolytic therapy for calcified cerebral emboli originated from calcified internal carotid artery stenosis. Rinsho Shinkeigaku. (2009) 49:281–4. doi: 10.5692/clinicalneurol.49.281 19594108

[36] O’Cearbhaill R Moriarty H Crosbie I Gd K Bolster F Ao H. Calcified cerebral emboli: a case series and review of literature. J Syst Integr Neurosci. (2016) 2:180–3. doi: 10.15761/JSIN.1000133

[37] Armstrong P Chiu AHY Phatouros CC. Calcified cerebral emboli: incidence and implications—how do you know an embolism is calcific? J Med Imaging Radiat Oncol. (2018) 62:814. doi: 10.1111/1754-9485.12806 30203920

[38] Koh E Kwak HS Chung GH. Manual aspiration Thrombectomy in patients with acute stroke-related calcified cerebral emboli. J Stroke Cerebrovasc Dis. (2017) 26:2050–4. doi: 10.1016/j.jstrokecerebrovasdis.2016.07.005, PMID: 28712720

[39] Yasaka M Tsuchiya T Yamaguchi T. Mobile string-like thrombus on the calcified aortic valve in cardioembolic stroke--a case report. Angiology. (1993) 44:655–9. doi: 10.1177/000331979304400811, PMID: 8342883

[40] Haboub M Abouradi S Mechal H Minko G Moukhliss A Arous S. Spontaneous calcific cerebral embolization revealing a calcified rheumatic mitral stenosis: a case report. J Med Case Rep. (2023) 17:254. doi: 10.1186/s13256-023-03982-2, PMID: 37330507 PMC10276911

[41] Gearry RB Sharr JP Avery SF. Spontaneous calcific cerebral embolus. Australas Radiol. (2005) 49:154–6. doi: 10.1111/j.1440-1673.2005.01361.x 15845055

[42] Oliveira-Filho J Massaro AR Yamamoto F Bustamante L Scaff M. Stroke as the first manifestation of calcific aortic stenosis. Cerebrovasc Dis. (2000) 10:413–6. doi: 10.1159/000016099, PMID: 10971029

[43] Xu SC Canter L Zeeshan A Elefteriades JA. Deep crater in heavily calcified aortic valve leaflet: a "smoking gun" for embolic stroke. Aorta. (2015) 3:172–6. doi: 10.12945/j.aorta.2015.15.010, PMID: 27175368 PMC4851852

[44] Potts MB da Matta L Abdalla RN Shaibani A Ansari SA Jahromi BS. Stenting of Mobile calcified emboli after failed Thrombectomy in acute ischemic stroke: case report and literature review. World Neurosurg. (2020) 135:245–51. doi: 10.1016/j.wneu.2019.12.096, PMID: 31881346

[45] Giannopoulos S Speziale F Vadala G Soukas P Kuhn BA Stoltz CL. Intravascular lithotripsy for treatment of calcified lesions during carotid artery stenting. J Endovasc Ther. (2021) 28:93–9. doi: 10.1177/1526602820954244, PMID: 32869718

[46] Arboix A Garcia-Eroles L Sellares N Raga A Oliveres M Massons J. Infarction in the territory of the anterior cerebral artery: clinical study of 51 patients. BMC Neurol. (2009) 9:30. doi: 10.1186/1471-2377-9-30, PMID: 19589132 PMC2714497

