# Supplementary material for: Spontaneous calcified cerebral emboli: a comprehensive review and proposed diagnostic criteria
Source: Front Neurol. 2024 Jul 17;15:1401820. doi: 10.3389/fneur.2024.1401820 (PMC11288925; doi:10.3389/fneur.2024.1401820)
Supplement: Supplementary file 1 [file Table_1.DOCX]

**Supplementary Table 1.** Published cases of Spontaneous Calcified Cerebral Emboli from Calcified Aortic Valves.

| **Source** | **Patient age and gender** | **Presumed embolic source** | **Cerebral vessel affected** | **Number of emboli** | **Treatment received** | **Outcome** |
| --- | --- | --- | --- | --- | --- | --- |
| Yock et al., 1981, Case 3 | 57M | Calcified aortic valve | Right and left MCA | 2 | Heparin therapy, discharged on coumadin | Rapid recovery |
| Rancurel et a1., 1989 | 73M | Calcified aortic stenosis | Left MCA (M2) | 1 | Heparin therapy,  Aortic valvular replacement | Mild semantic impairment and slow writing ability |
| Vernhet et al., 1993 | 2 patients, age/gender not specified | Aortic valve origin for both patients | Not specified | Not specified | Not specified | Not specified |
| Yasaka et al., 1993 | 72F | Calcified aortic stenosis | Right MCA | 1 | Antiplatelet therapy | Recurrence of cerebral embolism 3 months after discontinuation of antiplatelet therapy |
| O’Donoghue et al., 1993 | 36M | Calcified bicuspid aortic valve | Right MCA | 2 | Heparin therapy,  Aortic valvular replacement | Movement regained in left leg but the left face and arm weakness remained unchanged. |
| Doiron et al., 1996 | 47M | Calcified bicuspid aortic valve | Right MCA | 1 | Aortic valvular replacement | Complete recovery 1 year later |
| Shanmugan et al., 1997 | 40M | Calcified bicuspid aortic valve | Right MCA (M1) | 1 | Aortic valvular replacement | Complete recovery 4 weeks later |
| Oliveira-Filho et al., 2000 | 46M | Calcified aortic stenosis | Right MCA | 1 | Heparin and coumadin therapy,  Aortic valvular replacement,  Long term antiplatelet therapy | Silent myocardial infarction 2 months after initial event, presumed to be also embolic in origin from the calcified aortic stenosis. Following aortic valve replacement, the patient has been symptom-free (no further strokes) during a 2-year follow-up. |
| Kissela et al., 2001, Case 2 | 80F | Calcified aortic valve | Right MCA (M1) and basilar artery | 2 | IV thrombolytic therapy | Death |
| Martínez-Fernández et al., 2002, Case 2 | 47F | Severe aortic valve calcification and considerable mitral valve annulus calcification | Left MCA (M1) | 1 | Not specified | Not specified |
| Debruxelles et al., 2004, Case 1 | 56F | Calcified aortic stenosis | Left and right MCA | 2 | Aortic valve replacement | The motor deficit resolved spontaneously within a few days and only the aphasia persisted |
| Debruxelles et al., 2004, Case 2 | 57M | Calcified aortic stenosis | Right MCA and PCA | 2 | Aortic valve replacement | Partial recovery of the motor deficit |
| Gearry et al., 2005 | 29M | Calcified bicuspid aortic valve with stenosis | Left MCA (M1) | 1 | Heparin infusion,  Aortic valve replacement | Mild right sided weakness when fatigued |
| Bugnicourt et al., 2008 | 81M | Calcified aortic stenosis | Distal segment of left ACA | 1 | Antiplatelet agents and systematic antihypertensive treatment instituted,  Indication for aortic valve replacement explained but patient refused. | Disappearance of motor symptoms in a few days after treatment but persistence of dysarthria |
| Roifman et al., 2009 | 40M | Calcified aortic valve | Right MCA | 1 | Heparin and low dose aspirin,  Aortic valve replacement | Uncomplicated postoperative course and was discharged home |
| Yong et al., 2010 | 54M | Heavily calcified aortic valve and ascending aorta atheroma | Left MCA | 2 | Not specified | Not specified |
| Walker et al., 2014, Case 6 | 20M | Aortic stenosis | Right and left MCA | 6 | Aortic valve replacement | Residual impairment |
| Walker et al., 2014, Case 9 | 83M | Aortic stenosis | Right MCA | 6 | Not specified | Residual impairment |
| Walker et al., 2014, Case 15 | 68M | Aortic stenosis | Right MCA | 1 | Aortic valve replacement | Recovery |
| Walker et al., 2014, Case 16 | 59M | Aortic stenosis | Right MCA | 1 | Not specified | Death |
| Walker et al., 2014, Case 17 | 73F | Aortic stenosis | Right MCA | 1 | Aortic valve replacement | Residual impairment |
| Walker et al., 2014, Case 18 | 73M | Aortic stenosis | Right PCA | 1 | IV thrombolytic therapy | Recovery |
| Walker et al., 2014, Case 20 | 79M | Aortic stenosis | Left MCA | 2 | Not specified | Recovery |
| Kobayashi 2017 | 80F | Calcified aortic valves | Left MCA and ACA | 16 | IV edaravone and glycerol | Severe neurological deficits remain |
| Thunstedta et al., 2020 | 29M | Calcified bicuspid aortic valve | Right MCA (M2) | 1 | IV thrombolytic therapy, Acetylsalicylic acid, statin, new oral anticoagulant therapy,  Aortic valve replacement | Persistent mild fine-motor disturbance in the left hand |
| Taoussi et al., 2022 | 59M | Calcified aortic and mitral stenosis | Right and left MCA (M3, M4) and distal branches of the posterior arteries | Many (exact amount not specified) | Antiplatelet therapy | Excellent recovery in both right upper and lower limb power after a few days |
| Mosqueira et al., 2022 | 2 patients, age/gender not specified | Sclerotic and calcified aortic valve | Not specified | Not specified | Not specified | Not specified |
| Mahajan et al., 2022 | 54M | Calcified aortic stenosis | Right MCA (M1) | 1 | IV thrombolytic therapy, endovascular clot retrieval | Minimal residual left hemiparesis |

Acronyms: F – Female, M – Male, ACA – Anterior Cerebral Artery, MCA – Middle Cerebral Artery, PCA – Posterior Cerebral Artery, IV - Intravenous.
